# Retraction: Dexamethasone Rescues Neurovascular Unit Integrity from Cell Damage Caused by Systemic Administration of Shiga Toxin 2 and Lipopolysaccharide in Mice Motor Cortex

**DOI:** 10.1371/journal.pone.0295781

**Published:** 2023-12-07

**Authors:** The PLOS ONE Editors

Following the publication of this article [1], concerns were raised regarding results presented in Figs 3, 4, 5, 6, 7, and 8. Specifically,

The following panels appear similar, despite being used to represent different experimental conditions:
  ○ Fig 3A and Fig 4F (Control 2 days and LPS Dexamethasone respectively)
  ○ Fig 3B and Fig 4H (LPS 2 days and Stx2+LPS Dexamethasone respectively)
  ○ Fig 3D, Fig 3F, and Fig 4C (Stx2+LPS 2 days, LPS 4 days, and Stx2 Without Dexmethasone respectively)
  ○ Fig 7L and Fig 8D (Stx2+LPS 7 days and Stx2+LPS Without Dexamethasone respectively)
  ○ Fig 7P and Fig 8C (Stx2+LPS 20 days and Stx2 Without Dexamethasone respectively)
The following panels appear to partially overlap, despite being used to represent different experimental conditions:
  ○ Fig 3I and Fig 3M (Control 7 days and Control 20 days respectively)
  ○ Fig 5O and Fig 5P (Stx2 20 days and Stx2+LPS 20 days respectively)
  ○ Fig 6A and Fig 6E (Control Without Dexamethasone and Control Dexamethasone respectively)
Fig 4J and Fig 6J appear similar.

The corresponding author stated that the panel duplications are the result of inadvertent errors during figure preparation. The original images underlying these results are no longer available. In the absence of the underlying data, the concerns listed in this notice cannot be resolved.

The extent of the image concerns observed in this article raise serious concerns regarding the handling of the data obtained during this study and the overall reliability of the published results. In light of these concerns the *PLOS ONE* Editors retract this article.

AP and JG did not agree with the retraction and stand by the article’s findings. MJ, PAG, AC, MLC, and CTF either did not respond directly or could not be reached.
